# A multimodal ultrasound-based model combining tumor radiomics and axillary lymph node morphologic classification for predicting axillary nodal burden in breast cancer

**DOI:** 10.3389/fendo.2026.1847368

**Published:** 2026-06-04

**Authors:** Renjie Lu, Taiyu Yang, Linlin Pan, Linlin Shao, Lili Zhang, Fangfang Sun, Jie Du, Lirong Zhao

**Affiliations:** 1 Department of Radiology, The First Hospital of Jilin University, Changchun, Jilin, China; 2 Ultrasound Diagnostic Center, The First Hospital of Jilin University, Changchun, Jilin, China

**Keywords:** axillary lymph node burden, breast cancer, machine learning, radiomics, ultrasound

## Abstract

**Objective:**

To develop and validate a multimodal ultrasound-based model integrating tumor radiomics and axillary lymph node (ALN) morphologic classification for preoperative prediction of axillary nodal metastasis burden in breast cancer.

**Methods:**

This retrospective study included 583 patients with pathologically confirmed breast cancer, randomly divided into training and testing cohorts (7:3). Radiomic features were extracted from primary tumor ultrasound images, and ALN classification and clinicopathological variables were collected. A hierarchical modeling strategy was applied: first-level models predicted ALN metastasis (N_0_ vs. N_+_), and second-level models distinguished axillary nodal tumor burden (N_1–2_ vs. N_≥3_). Machine learning algorithms were used to construct radiomics models, and combined models were developed by integrating the radiomics score (Rad-score) with independent predictors. Model performance was evaluated using ROC analysis and decision curve analysis.

**Results:**

At the first level, the radiomics model achieved an AUC of 0.79, while the combined model incorporating ALN classification improved performance to 0.90. At the second level, the radiomics model yielded an AUC of 0.74, which increased to 0.78 after integration. Ki-67 was identified as an independent predictor. Subgroup analysis showed that the combined model performed consistently well in predicting ALN metastasis across Ki-67 subgroups, whereas its performance in distinguishing nodal burden was superior in the low Ki-67 subgroup (AUC 0.82 vs. 0.68).

**Conclusion:**

The proposed multimodal model enables accurate, noninvasive prediction of ALN metastasis and axillary nodal tumor burden. Integrating tumor radiomics with lymph node morphology may support individualized risk stratification and treatment planning and optimize axillary management.

## Introduction

Breast cancer remains the most commonly diagnosed malignancy among women worldwide (1). Notably, the incidence of breast cancer has been increasing more rapidly in women younger than 50 years compared with those aged 50 years or older over the past decade (1, 2). Accurate assessment of axillary lymph node (ALN) involvement is essential for disease staging, prognostic stratification, treatment decision-making and quality of life in patients with breast cancer, particularly for young women (3, 4). Historically, mastectomy combined with axillary lymph node dissection (ALND) was considered the standard surgical approach for breast cancer (3). However, emerging evidence, including the SENOMAC trial, supports the safe omission of ALND in selected patients with limited nodal burden (5). Consequently, patients can be stratified into three clinically relevant subgroups based on axillary nodal tumor burden: node-negative (N_0_), low nodal burden (1–2 positive nodes), and high nodal burden (≥3 positive nodes). Accurate preoperative assessment of ALN status is therefore critical for guiding adjuvant therapy and determining the need for ALND. The sentinel lymph node (SLN), defined as the first node receiving lymphatic drainage from the primary tumor, serves as a key indicator of axillary status (6). Sentinel lymph node dissection (SLND) remains the gold standard for axillary staging, particularly in clinically node-negative patients (4). However, SLND is still an invasive procedure and may lead to complications such as numbness, lymphedema, and impaired arm mobility, affecting approximately 3%–11% of patients (7, 8). Moreover, studies have shown that 40%–60% of patients with positive SLNs have no additional non-sentinel lymph node metastases, potentially resulting in overtreatment and unnecessary axillary surgery (9, 10).

Despite advances in imaging techniques, accurate preoperative assessment of ALN status, particularly axillary nodal tumor burden, remains challenging. Ultrasound is widely used in clinical practice due to its accessibility, cost-effectiveness, and non-invasive nature, and plays an important role in the evaluation of breast lesions and axillary lymph nodes (11). However, conventional axillary ultrasound relies heavily on operator-dependent qualitative assessment (12). In addition, its sensitivity remains limited leading to a non-negligible false-negative rate (13, 14). In recent years, radiomics approaches have shown great potential in improving the prediction of ALN metastasis by extracting high-dimensional quantitative features from ultrasound images of breast lesions (15, 16). Several studies have demonstrated that ultrasound-based radiomics models, particularly when combined with immunohistochemistry variables, can effectively predict ALN status (16–18). However, most existing studies primarily focus on binary outcomes, distinguishing between metastatic and non-metastatic cases, and typically rely on imaging features derived from the primary tumor alone, with limited integration of ALN-specific imaging characteristics. From a pathophysiological perspective, metastatic deposits initially occur in the cortical region of lymph nodes and lead to progressive morphologic alterations, which can be detected by ultrasound (19, 20). A classical sonographic classification based on cortical morphologic features (types 1-6) has been proposed, in which asymmetric focal cortical lobulation (type 5) and complete loss of the fatty hilum (type 6) are strongly associated with metastatic involvement, whereas types 1–4 are generally indicative of benign findings (19). Integrating primary tumor imaging features with ALN morphologic classification may enable the development of a more biologically interpretable and clinically relevant model for preoperative prediction of axillary nodal tumor burden.

Therefore, this study aimed to develop and validate a multimodal ultrasound-based model integrating primary tumor radiomics features and ALN morphologic classification, along with conventional ultrasound and immunohistochemistry variables, for preoperative prediction of axillary nodal tumor burden, enabling three-category stratification [N_0_, N_1–2_ (low burden: 1–2 positive nodes) and N_≥3_ (high burden: ≥3 positive nodes)] in patients with breast cancer.

## Methods

**Figure 1:**
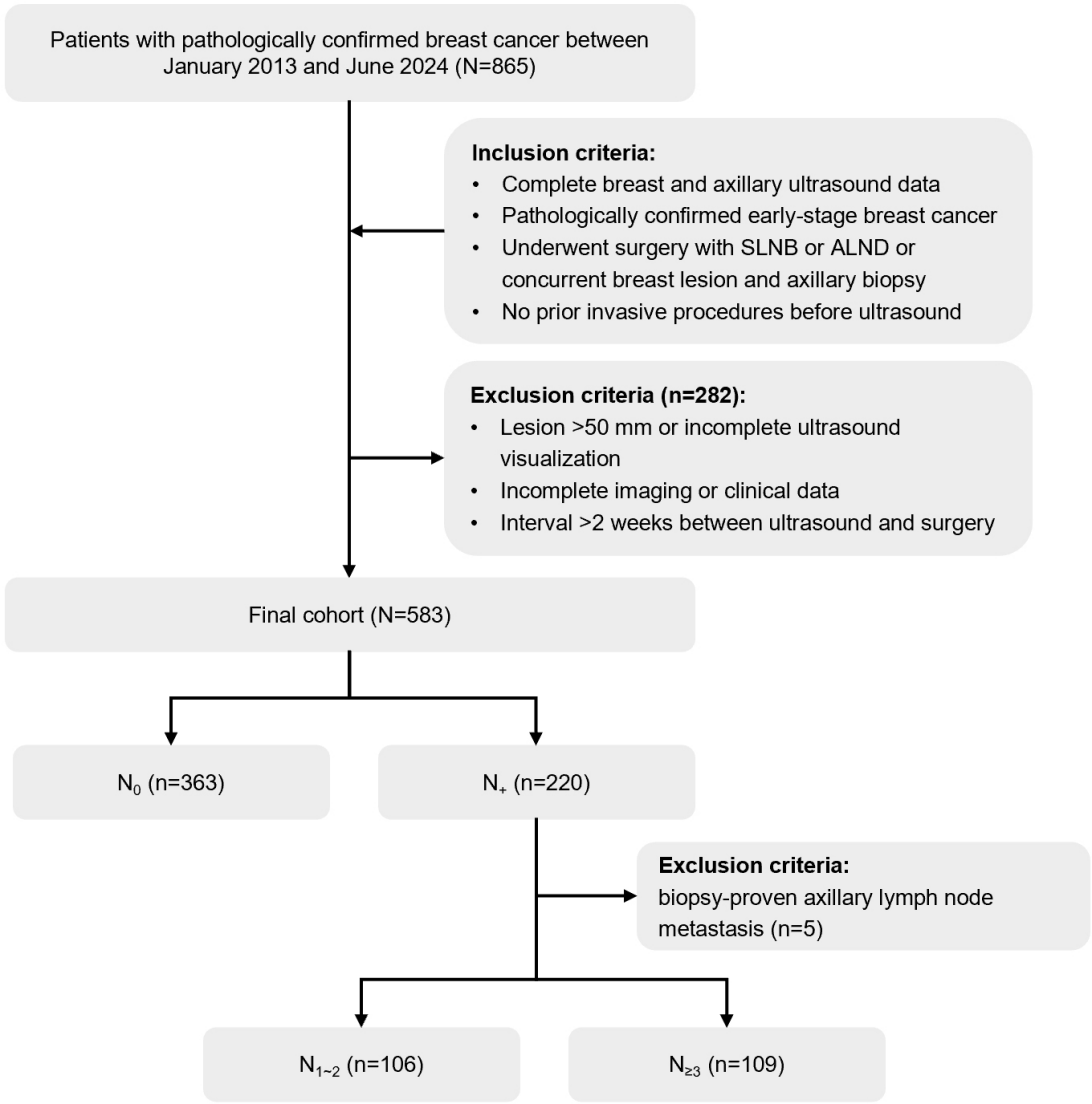
Study flowchart of patient selection and cohort construction. A total of 865 patients with pathologically confirmed breast cancer were initially screened. After applying the inclusion and exclusion criteria, 583 patients were finally enrolled. The cohort was divided into N_0_ (no axillary lymph node metastasis, n = 363) and N_+_ (presence of metastasis, n = 220). After excluding patients with biopsy-proven axillary lymph node metastasis (n = 5), the N_+_ group was further stratified into low nodal burden (N_1–2_, n = 106) and high nodal burden (N_≥3_, n = 109).

This retrospective study was approved by the Ethics Committee of the First Hospital of Jilin University, and the requirement for informed consent was waived due to its retrospective design (2024–1266). Consecutive patients with pathologically confirmed breast cancer between January 2013 and June 2024 were screened (**Figure 1**). Ultrasound images, clinical data, and immunohistochemistry data were collected for all patients. The inclusion criteria were as follows: (1) pathologically confirmed early-stage breast cancer; (2) complete breast and axillary ultrasound data; (3) patients who underwent surgery with SLNB or ALND, or concurrent breast lesion and ALN biopsy; and (4) no prior invasive procedures (e.g., biopsy or neoadjuvant therapy) before ultrasound examination. The exclusion criteria were as follows: (1) lesion diameter >50 mm or incomplete ultrasound visualization; (2) incomplete imaging or clinical data; (3) an interval >2 weeks between ultrasound examination and surgery; and (4) biopsy-proven ALN metastasis prior to surgery, which precluded accurate assessment of axillary nodal tumor burden. All surgical specimens were independently evaluated by two experienced pathologists. Immunohistochemistry parameters included histological type, estrogen receptor (ER), progesterone receptor (PR), human epidermal growth factor receptor 2 (HER2), and Ki-67 index. Axillary nodal status was determined based on postoperative histopathology and categorized according to the number of metastatic lymph nodes into three groups: node-negative (N_0_), low nodal burden (N_1–2_), and high nodal burden (N_≥3_).

### Ultrasound image acquisition and analysis

All ultrasound examinations were performed using two Philips EPIQ 5 color Doppler ultrasound systems and a Philips iU22 color Doppler ultrasound system (Philips Healthcare, Andover, MA, USA) equipped with high-frequency linear-array transducers operating at 6–18 MHz. Patients were examined in the supine position, and excessive transducer pressure was avoided to minimize distortion of the lesion and surrounding tissues. A comprehensive bilateral breast and axillary ultrasound examination was conducted preoperatively by 5–6 experienced radiologists. All ultrasound images were independently re-reviewed by two radiologists with 5 and 10 years of experience in breast imaging, respectively. Both radiologists were blinded to the other clinical information. The following ultrasound features were recorded for each patient: Breast Imaging Reporting and Data System (BI-RADS) category, lesion size (maximum length and width), lesion position, ALN status, lymph node size (maximum length and width), and lymph node classification based on morphologic characteristics (19). Background parenchymal echotexture was also assessed using the glandular tissue component (GTC) (21). In addition, a history of contralateral breast cancer was documented. For patients with multiple lesions in the ipsilateral breast, only the largest and most suspicious lesion was selected for analysis.

Ultrasound images were imported into 3D Slicer software (version 5.0.2) for lesion segmentation. Regions of interest (ROIs) of two-dimensional grayscale images were manually delineated using the “Segment Editor” module. Two radiologists, who were blinded to the patients’ other information, independently performed the ROI segmentation. In cases of disagreement, a senior radiologist reviewed the images and provided the final delineation.

### Feature extraction and selection

Radiomic feature extraction was performed using the Radiomics module implemented in 3D Slicer, which is based on the open-source PyRadiomics library. For each ROI, radiomic features were extracted from both original and filtered images. All features were extracted in accordance with the Image Biomarker Standardization Initiative guidelines.

All patients were randomly divided into a training cohort and a testing cohort at a ratio of 7:3. Within the training cohort, an initial feature screening was performed using the independent two-sample t-test, and features with a *P* value < 0.05 were retained for further analysis. Subsequently, dimensionality reduction was conducted using the least absolute shrinkage and selection operator (LASSO) regression with 10-fold cross-validation to identify the most predictive features. Finally, a radiomics model was constructed based on the selected features.

### Model development and validation

Model development and validation were performed in a hierarchical manner. At the first level, models were constructed to discriminate between patients with and without ALN metastasis (N_0_ vs. N_+_). At the second level, models were further developed to distinguish low nodal burden (N_1–2_) from high nodal burden (N_≥3_). At each level, two types of models were established: a radiomics model and a combined model integrating radiomics and ultrasound features. Four machine learning algorithms were evaluated, including logistic regression (LR), support vector machine (SVM), random forest (RF), and extreme gradient boosting (XGBoost, XGB). The best-performing model was selected, and its predicted probability was defined as the radiomics score (Rad-score), which was subsequently used as a key variable for further analysis. SHAP (SHapley Additive exPlanations; version 0.41) analysis was used to evaluate feature contributions in the optimal model, quantifying their impact and visualizing their importance in predicting ALN metastasis risk. The combined model was constructed by integrating the Rad-score with ultrasound features. Independent predictors were identified using univariable and multivariable logistic regression analyses, and a final predictive model was established accordingly. Finally, the developed models were visualized as nomograms to provide an intuitive and quantitative tool for preoperative assessment of ALN metastasis in patients with breast cancer. The optimal cutoff value for Ki-67 was determined using the Youden index (*sensitivity + specificity - 1*) in the training cohort. Subgroup analysis was then performed by stratifying patients according to this cutoff value.

### Statistical analysis

All statistical analyses were performed using Python (version 3.11.5) and R statistical software (version 4.3.2). Continuous variables were compared using the independent-sample t-test or Mann–Whitney U test, as appropriate, while categorical variables were compared using the chi-square (χ²) test. The development and validation of the machine learning models were conducted using the scikit-learn library in Python. Model performance was evaluated by receiver operating characteristic (ROC) curve analysis, and the area under the curve (AUC) was calculated to assess discriminative ability in the testing cohort. Decision curve analysis (DCA) was performed to evaluate the clinical utility of the models. The DeLong test was used to compare differences in AUCs between models. *P* value < 0.05 was considered statistically significant.

## Results

### Patient population

A total of 583 patients were included in this study (**Figure 1**). Patients were randomly assigned to a training cohort (n = 408) and a testing cohort (n = 175) at a ratio of 7:3. ALN metastasis was present in 220 patients (37.7%) and not detected in 363 (62.3%). Among metastatic cases, patients were further categorized into low nodal burden (N_1–2_, n = 106) and high nodal burden (N_≥3_, n = 109). No significant differences were observed between the training and testing cohorts in baseline characteristics, indicating good comparability between the two datasets. (**Table 1**). Baseline characteristics stratified by nodal burden (N_1–2_ vs. N_≥3_) are provided in **Supplementary Table 1**, showing consistent distributions across cohorts.

**Table 1:** Clinicopathologic and ultrasound characteristics of patients in the training and test cohorts.

Variable	Category	Train (N=408)			Test (N=175)		
		N_0_ (N=256)	N_+_ (N=152)	p	N_0_ (N=107)	N_+_ (N=68)	p
Age		53.9 ± 10.9	53.2 ± 9.8	0.475	54.2 ± 10.8	55.8 ± 10.7	0.322
Molecular subtype				0.010			0.088
	Luminal A	93 (36.3%)	35 (23%)		37 (34.6%)	16 (23.5%)	
	Luminal B1	31 (12.1%)	36 (23.7%)		14 (13.1%)	15 (22.1%)	
	Luminal B2	79 (30.9%)	49 (32.2%)		32 (29.9%)	25 (36.8%)	
	HER2 overexpression	27 (10.5%)	16 (10.5%)		10 (9.3%)	9 (13.2%)	
	Triple-Negative	26 (10.2%)	16 (10.5%)		14 (13.1%)	3 (4.4%)	
ER		0.6 ± 0.4	0.5 ± 0.4	0.092	0.6 ± 0.4	0.7 ± 0.4	0.495
PR		0.5 ± 0.4	0.4 ± 0.4	0.036	0.5 ± 0.4	0.5 ± 0.4	0.787
HER2				0.990			0.221
	0	115 (44.9%)	67 (44.1%)		43 (40.2%)	27 (39.7%)	
	1	34 (13.3%)	22 (14.5%)		23 (21.5%)	7 (10.3%)	
	2	63 (24.6%)	37 (24.3%)		25 (23.4%)	20 (29.4%)	
	3	44 (17.2%)	26 (17.1%)		16 (15%)	14 (20.6%)	
Ki-67		0.3 ± 0.2	0.4 ± 0.2	0.001	0.3 ± 0.2	0.4 ± 0.2	0.111
AUS report							<0.001
	Suspicious	31 (12.1%)	106 (69.7%)		10 (9.3%)	41 (60.3%)	
	Unsuspicious	225 (87.9%)	46 (30.3%)	<0.001	97 (90.7%)	27 (39.7%)	
BI-RADS				<0.001			<0.001
	3	14 (5.5%)	6 (3.9%)		11 (10.3%)	2 (2.9%)	
	4a	53 (20.7%)	6 (3.9%)		24 (22.4%)	1 (1.5%)	
	4b	57 (22.3%)	18 (11.8%)		30 (28%)	10 (14.7%)	
	4c	93 (36.3%)	70 (46.1%)		31 (29%)	28 (41.2%)	
	5	39 (15.2%)	52 (34.2%)		11 (10.3%)	27 (39.7%)	
Lesions size							
	Length	2.0 ± 1.2	2.5 ± 1.3	<0.001	1.8 ± 1.1	2.8 ± 1.2	<0.001
	Width	1.3 ± 0.8	1.7 ± 0.9	<0.001	1.2 ± 0.6	1.8 ± 0.8	<0.001
ALN size							
	Length	0.5 ± 0.9	1.4 ± 1.0	<0.001	0.4 ± 0.7	1.4 ± 1.2	<0.001
	Width	0.2 ± 0.4	0.8 ± 0.6	<0.001	0.1 ± 0.3	0.7 ± 0.6	<0.001
GTC				0.766			0.041
	1	141 (55.1%)	83 (54.6%)		58 (54.2%)	50 (73.5%)	
	2	80 (31.2%)	53 (34.9%)		35 (32.7%)	13 (19.1%)	
	3	26 (10.2%)	12 (7.9%)		6 (5.6%)	4 (5.9%)	
	4	9 (3.5%)	4 (2.6%)		8 (7.5%)	1 (1.5%)	
ALN Type				<0.001			<0.001
	0	183 (71.5%)	33 (21.7%)		82 (76.6%)	21 (30.9%)	
	1	7 (2.7%)	2 (1.3%)		6 (5.6%)	2 (2.9%)	
	2	37 (14.5%)	5 (3.3%)		9 (8.4%)	1 (1.5%)	
	3	15 (5.9%)	8 (5.3%)		6 (5.6%)	5 (7.4%)	
	4	9 (3.5%)	5 (3.3%)		1 (0.9%)	2 (2.9%)	
	5	2 (0.8%)	29 (19.1%)		2 (1.9%)	14 (20.6%)	
	6	3 (1.2%)	70 (46.1%)		1 (0.9%)	23 (33.8%)	
Lesion position							
The lateral breast				0.002			0.229
	involved	186 (72.7%)	131 (86.2%)		82 (76.6%)	58 (85.3%)	
	uninvolved	70 (27.3%)	21 (13.8%)		25 (23.4%)	10 (14.7%)	
The medial breast				0.012			0.714
	involved	125 (48.8%)	54 (35.5%)		42 (39.3%)	24 (35.3%)	
	uninvolved	131 (51.2%)	98 (64.5%)		65 (60.7%)	44 (64.7%)	
The inferior breast				<0.001			0.014
	involved	64 (25%)	63 (41.4%)		31 (29%)	33 (48.5%)	
	uninvolved	192 (75%)	89 (58.6%)		76 (71%)	35 (51.5%)	
The superior breast				0.279			0.065
	involved	228 (89.1%)	129 (84.9%)		98 (91.6%)	55 (80.9%)	
	uninvolved	28 (10.9%)	23 (15.1%)		9 (8.4%)	13 (19.1%)	
CBC history				0.199			0.651
	yes	6 (2.3%)	8 (5.3%)		6 (5.6%)	2 (2.9%)	
	no	250 (97.7%)	144 (94.7%)		101 (94.4%)	66 (97.1%)	

Data are presented as mean ± standard deviation or number (%). N₀, patients with negative axillary lymph node (ALN) metastasis; N, patients with positive ALN metastasis. AUS, axillary ultrasound; BI-RADS, Breast Imaging Reporting and Data System; ALN, axillary lymph node; GTC, glandular tissue component; CBC, contralateral breast cancer. P values were calculated using the independent-samples t-test for continuous variables or the chi-square test for categorical variables, comparing the ALN-negative and ALN-positive groups.

### Feature extraction and selection

A total of 851 radiomic features were extracted from ultrasound images of breast lesions. After initial screening (*P* < 0.05) and LASSO regression, 15 radiomic features were retained for model construction at the first level. For conventional ultrasound and clinicopathological variables, univariable and multivariable logistic regression analyses showed that ALN classification (19) as a significant ultrasound predictor and Ki-67 as the only independent immunohistochemical predictor at this level (**Supplementary Tables 2**, **3**). At the second level, feature selection resulted in 5 radiomic features. Multivariable analyses yielded consistent results, with ALN classification and Ki-67 identified as independent factors (**Supplementary Tables 4**, **5**).

### Model evaluation and interpretability

**Figure 2:**
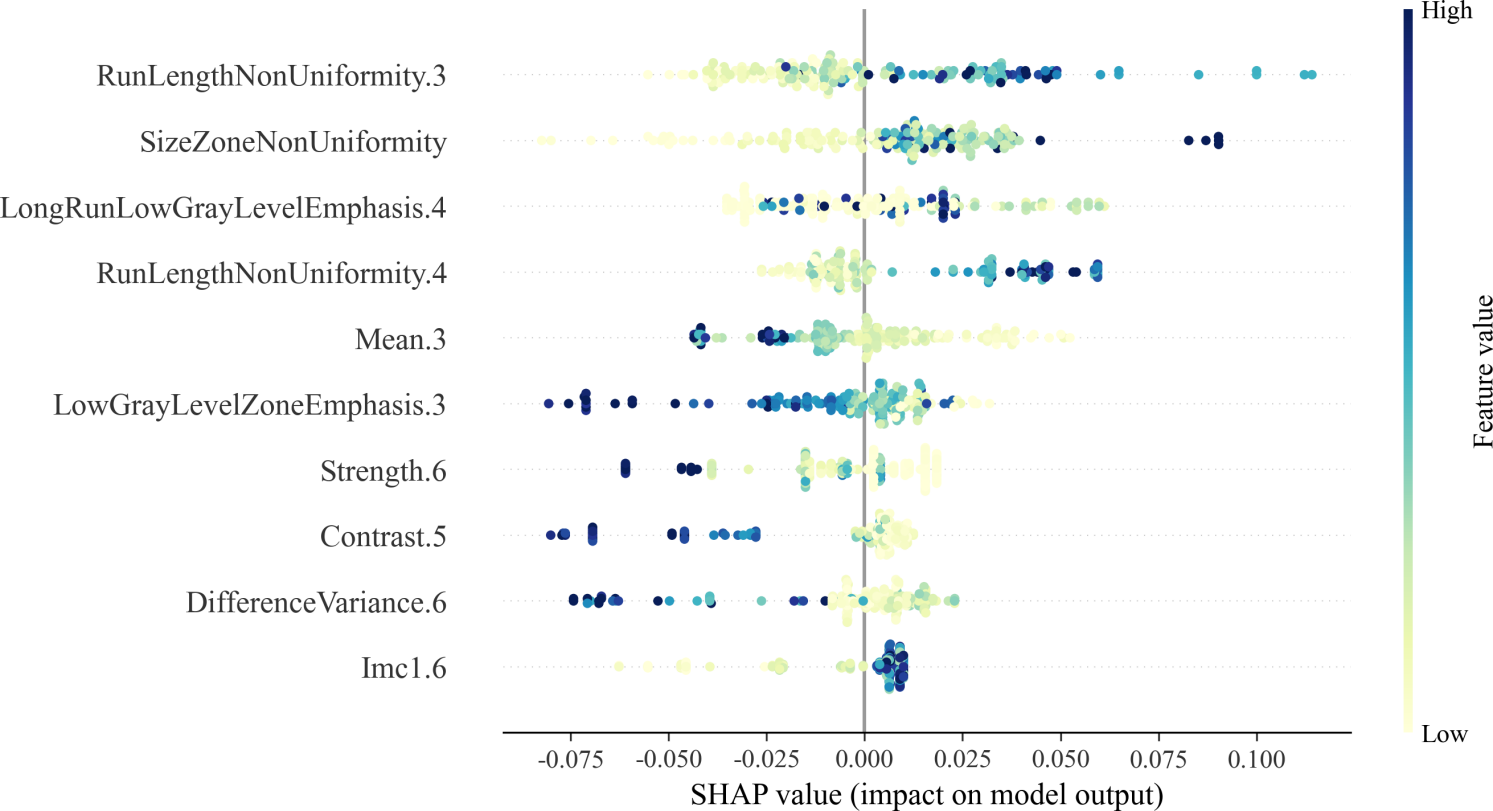
SHAP summary plot of the radiomics model for predicting axillary lymph node metastasis. The plot shows the top-ranked radiomic features based on their contribution to the model output. Each point represents an individual sample, with color indicating feature value (low to high). Features are ranked according to their importance, and the horizontal axis represents the SHAP value, reflecting the impact of each feature on model prediction.

**Figure 3:**
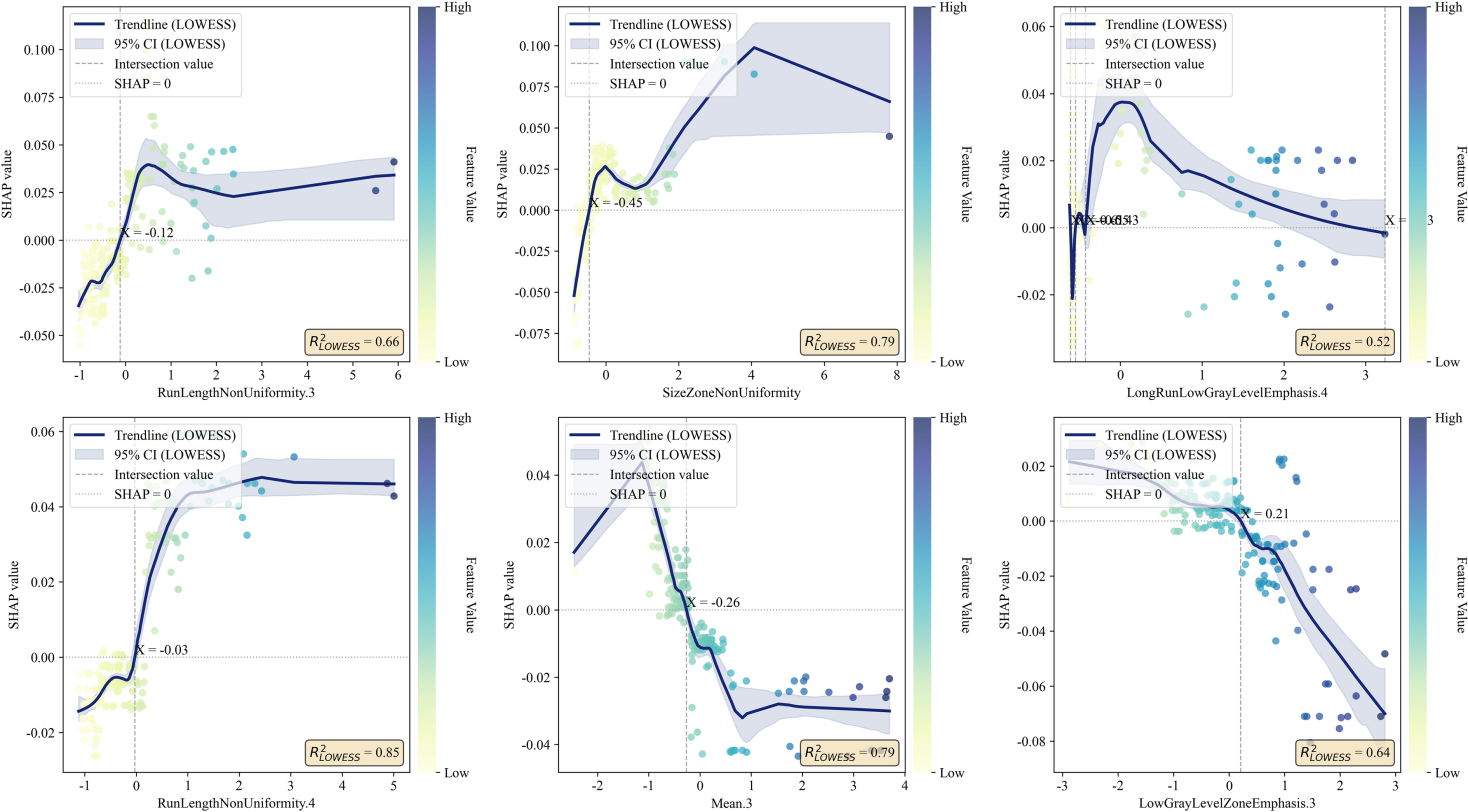
SHAP dependence plots of representative radiomic features for predicting axillary lymph node metastasis. The plots illustrate the relationship between selected radiomic features and their corresponding SHAP values. Nonlinear associations between feature values and predicted risk are observed, suggesting potential threshold effects. The solid line represents the locally weighted regression trend, and the shaded area indicates the 95% confidence interval.

**Figure 4:**
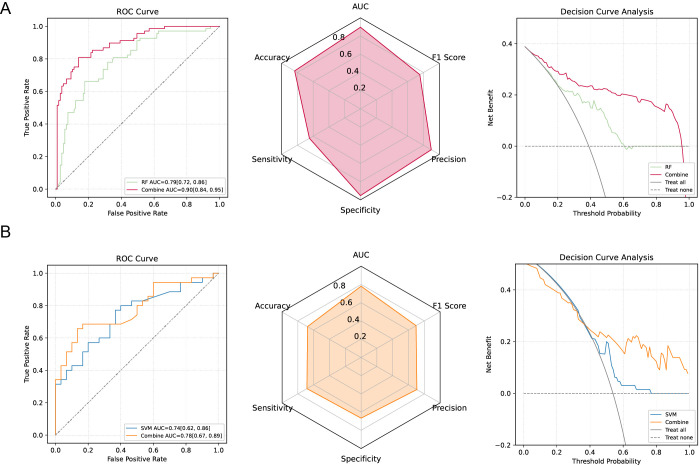
Diagnostic performance of the hierarchical models for predicting axillary lymph node burden. **(A)** Performance of the first-level model for predicting axillary lymph node metastasis (N_0_ vs. N_+_). Receiver operating characteristic (ROC) curves comparing the radiomics model (random forest) and the combined model; radar plot summarizing model performance (AUC, accuracy, sensitivity, specificity, precision, and F1 score); and decision curve analysis (DCA) showing the net clinical benefit across a range of threshold probabilities. **(B)** Performance of the second-level model for predicting axillary nodal tumor burden (N_1–2_ vs. N_≥3_). ROC curves comparing the radiomics model (support vector machine) and the combined model; radar plot summarizing model performance; and DCA demonstrating the net benefit across different threshold probabilities.

**Figure 5:**
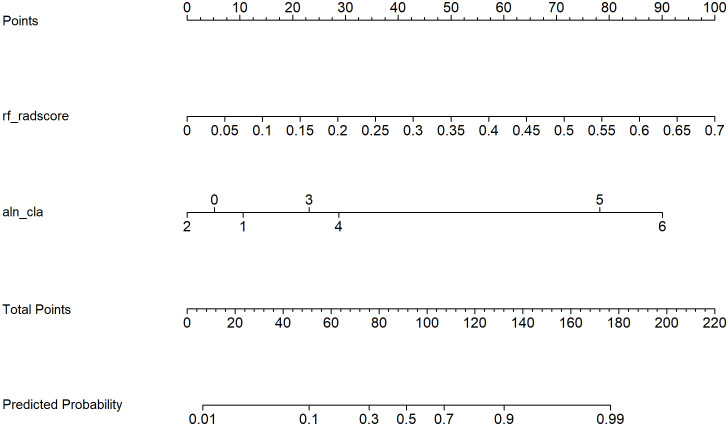
Nomogram for predicting the probability of axillary lymph node metastasis. The nomogram integrates the radiomics score (Rad-score) and axillary lymph node classification to estimate the probability of nodal metastasis. Each variable corresponds to a point value, and the total points are mapped to the predicted probability, providing an individualized and quantitative risk assessment.

For the prediction of ALN metastasis, the radiomics model achieved its best performance using the RF algorithm, with an AUC of 0.79 in the testing cohort (**Table 2**). The 10 most influential features in the radiomics model for predicting ALN metastasis (RF) are shown in **Figure 2**, with RunLengthNonUniformity and SizeZoneNonUniformity ranking highest. SHAP summary plots visualized global feature contributions, including both magnitude and direction. Dependence plots for the top six features (**Figure 3**) revealed non-linear relationships with predicted metastasis risk: higher values of heterogeneity-related features (e.g., RunLengthNonUniformity and SizeZoneNonUniformity) were associated with increased risk, whereas lower mean intensity values tended to indicate higher probability of metastasis. Other features showed similar nonlinear patterns with distinct threshold effects. For the combined model, only ALN classification and the Rad-score were retained for model construction. This model showed improved performance, achieving an AUC of 0.90 in the testing cohort, along with higher accuracy and specificity compared with the radiomics-only model (**Figure 4A**). The nomogram based on ALN classification and Rad-score (**Figure 5**) provides an individualized and quantitative tool for estimating the probability of ALN metastasis.

**Table 2:** Diagnostic performance of radiomics and combined models for predicting axillary lymph node metastasis (N_0_ vs. N_+_) at the first level.

Cohort	Model	AUC (95% CI)	Accuracy	Sensitivity	Specificity	P-value
Train						
	RF	0.790 (0.742,0.838)	0.708	0.830	0.630	<0.001
	Combine	0.913 (0.881,0.946)	0.860	0.691	0.961	<0.001
Test						
	RF	0.793 (0.721,0.865)	0.714	0.809	0.561	<0.001
	Combine	0.897 (0.844,0.950)	0.834	0.647	0.953	<0.001

AUC, area under the receiver operating characteristic curve; CI, confidence interval; RF, random forest; Combine, combined model integrating radiomics score and axillary lymph node (ALN) classification.

For the prediction of axillary nodal tumor burden, the radiomics model achieved its best performance using the SVM algorithm, with an AUC of 0.74 in the testing cohort (**Table 3**). The combined model further improved performance, achieving an AUC of 0.78 as shown in **Figure 4B**. Feature importance and SHAP analyses for the axillary nodal tumor burden model are presented in **Supplementary Figure 1**. Higher Ki-67 expression was associated with an increased risk of ALN metastasis and greater axillary nodal tumor burden.

**Table 3:** Diagnostic performance of radiomics and combined models for predicting nodal tumor burden (N_1–2_ vs. N_≥3_) at the second level.

Cohort	Model	AUC (95% CI)	Accuracy	Sensitivity	Specificity	P-value
Train						
	SVM	0.814 (0.745, 0.883)	0.713	0.851	0.579	<0.001
	Combine	0.885 (0.829, 0.940)	0.813	0.797	0.829	<0.001
Test						
	SVM	0.742 (0.623, 0.861)	0.662	0.829	0.467	<0.001
	Combine	0.780 (0.669, 0.892)	0.677	0.686	0.667	<0.001

AUC, area under the receiver operating characteristic curve; CI, confidence interval; SVM, support vector machine; Combine, combined model integrating radiomics score and axillary lymph node (ALN) classification.

The optimal cutoff value for Ki-67 determined by the Youden index was 0.45 for two levels. The performance of the combined model in different Ki-67 subgroups is summarized in **Table 4**. For predicting ALN metastasis (N_0_ vs. N_+_), the model demonstrated consistently high performance across both high and low Ki-67 groups in the testing cohorts (AUC: 0.892 vs. 0.885). For predicting nodal tumor burden (N_1–2_ vs. N_≥3_), the model achieved better performance in the low Ki-67 subgroup compared with the high Ki-67 subgroup in the testing cohort (AUC: 0.817 vs. 0.682).

**Table 4:** Subgroup analysis of model performance stratified by Ki-67 levels for predicting axillary nodal status and tumor burden.

Ki-67 subgroup	Cohort	AUC (95% CI)	Accuracy	Sensitivity	Specificity
N_0_ vs. N_+_					
Ki-67>0.45	Train	0.937 (0.892, 0.981)	0.877	0.841	0.910
	Test	0.892 (0.793, 0.992)	0.813	0.714	0.889
Ki-67<0.45	Train	0.912 (0.869, 0.954)	0.856	0.640	0.958
	Test	0.885 (0.818, 0.952)	0.811	0.574	0.950
N_1–2_ vs. N_≥3_					
Ki-67>0.45	Train	0.917 (0.847, 0.987)	0.833	0.842	0.818
	Test	0.682 (0.459, 0.905)	0.609	0.636	0.583
Ki-67<0.45	Train	0.865 (0.782, 0.948)	0.811	0.778	0.833
	Test	0.817 (0.690, 0.944)	0.738	0.708	0.778

Performance of the combined model in different Ki-67 subgroups for predicting axillary lymph node (ALN) metastasis (N0 vs. N+) and nodal tumor burden (N1–2 vs. N≥3). Patients were stratified into high and low Ki-67 groups using a cutoff value of 0.45. Model performance was evaluated in both the training and testing cohorts using the area under the receiver operating characteristic curve (AUC), accuracy, sensitivity, and specificity. Values in parentheses represent 95% confidence intervals.

## Discussion

This study aimed to develop a framework that integrates primary tumor radiomics features with ALN morphologic classification for the preoperative prediction of axillary nodal tumor burden in breast cancer. Unlike previous studies that mainly focused on radiomics features derived from the primary tumor alone, our approach incorporates lymph node–specific morphologic characteristics, enabling a more comprehensive and biologically interpretable assessment of axillary status. This combined strategy highlights the complementary value of integrating tumor radiomics with nodal features and may improve preoperative risk stratification and surgical decision-making in breast cancer.

Intratumoral heterogeneity is widely recognized as a key biological hallmark associated with tumor aggressiveness and lymph node metastasis in breast cancer. Increasing evidence indicates that spatial and microstructural heterogeneity reflects underlying variations in cellular density, stromal composition, angiogenesis, and necrosis, all of which contribute to tumor progression and metastatic dissemination (15, 22). These biological processes have been consistently highlighted in recent studies on breast cancer pathology, emphasizing the critical role of tumor microenvironment remodeling in driving metastatic potential (23, 24).Although these characterizations of tumor microstructure are subtle and visually imperceptible, radiomics captures them through texture features reflecting spatial heterogeneity, first-order intensity features describing global signal characteristics, and higher-order structural descriptors capturing tissue organization. Recent advances in radiomics have further demonstrated that such quantitative features can serve as imaging surrogates of tumor biology, bridging the gap between imaging phenotypes and histopathological characteristics (25, 26). Moreover, previous studies have shown that, across different ultrasound acquisition settings (including variations in transducer frequency), the stability and reproducibility of radiomic features can be improved through feature standardization and harmonized extraction protocols (27, 28). In the present study, texture features such as RunLengthNonUniformity and SizeZoneNonUniformity reflect variability in gray-level patterns, whereas Busyness characterizes the rate of local intensity change, together representing the degree of microstructural disorganization within the tumor. Elevated values of these features generally indicate increased heterogeneity, which has been associated with aggressive tumor phenotypes and a higher likelihood of lymphatic dissemination (29). In addition, first-order features such as Mean describe global intensity characteristics, with lower values potentially reflecting increased cellular density and more aggressive tumor behavior. Shape-related features, including Maximum3DDiameter, provide complementary information on tumor growth patterns, with larger tumors associated with a higher likelihood of lymph node metastasis and greater metastatic burden, consistent with the findings of previous reports (30). These findings provide a plausible explanation for the predictive performance of radiomics features, as they reflect underlying tumor heterogeneity associated with metastatic potential.

In addition to radiomics features, Ki-67 was also identified as an independent predictor, with higher expression associated with an increased risk of ALN metastasis and greater axillary nodal tumor burden, consistent with recent evidence (31). As a marker of cellular proliferation, Ki-67 reflects tumor growth kinetics and biological aggressiveness, which have been closely linked to metastatic potential (32, 33). However, its contribution to overall model performance was relatively limited compared with imaging-derived features. This may be because Ki-67 represents a single biological dimension, whereas radiomics and morphological features capture more comprehensive information regarding tumor heterogeneity and spatial organization. In the subgroup analysis, the proposed model demonstrated stable performance for predicting ALN metastasis across different Ki-67 levels, indicating its robustness in distinguishing node-negative and node-positive disease. However, for predicting nodal tumor burden, reduced performance was observed in the high Ki-67 subgroup. This may be attributed to increased intratumoral heterogeneity and more aggressive biological behavior in highly proliferative tumors, which could limit the discriminative ability of imaging-based features.

In contrast to other features that primarily reflect intrinsic tumor characteristics, ALN classification directly focuses on nodal involvement and provides additional information on tumor dissemination. In our study, ALN classification consistently served as a strong and stable predictor. Previous studies have shown that lymph node morphology reflects the extent of tumor infiltration within the lymphatic system. Morphological changes such as cortical thickening, loss of the fatty hilum, and alterations in nodal shape have been well established as imaging indicators of metastatic involvement (34, 35). However, in clinical practice, determining nodal metastasis is highly dependent on the clinician’s experience and remains challenging, particularly for assessing metastatic burden. ALN classification offers a more standardized approach and may better reflect the extent of nodal involvement. As shown in the nomogram, lower ALN categories (types 1-4) tend to be associated with non-metastatic status, further supporting its discriminative value (19). Therefore, incorporating ALN classification enables the model to capture not only tumor-intrinsic characteristics but also downstream manifestations of tumor spread.

While several studies have demonstrated that radiomics-based and deep learning models have good predictive value for ALN status in breast cancer, our model demonstrated competitive and robust performance. Zhang et al. reported that a radiomics model based on breast mass features achieved a relatively modest AUC of 0.745 for predicting ALN metastasis, whereas Wang et al. observed even lower performance (AUC = 0.647) (17, 36). In contrast, a deep learning radiomics model integrating conventional ultrasound and shear-wave elastography (SWE) achieved a higher AUC of 0.902 for ALN status prediction, which is comparable to our findings (9). For nodal tumor burden prediction, Wang et al. further reported an AUC of 0.820 using a combined clinical–radiomics model (37). Similarly, Wu et al. developed a multiclassifier radiomics model for predicting high ALN tumor burden, achieving AUCs ranging from 0.715 to 0.733 in the validation cohort (38). However, these studies may be limited by relatively small sample sizes or imbalanced cohort distributions. In comparison, our study incorporated multiple classifiers to construct a combined predictive model, developed a nomogram based on LR, and validated the results in an independent test cohort, thereby enhancing the robustness and reliability of the model. From a practical perspective, this approach is clinically convenient, as it requires only lesion delineation and ALN classification to estimate axillary nodal tumor burden. By integrating tumor intrinsic features with axillary status, the model provides a more comprehensive evaluation, which may help identify patients at higher risk of nodal involvement and guide individualized treatment strategies. In particular, it may assist in optimizing surgical decision-making and reducing unnecessary invasive procedures in patients with low metastatic risk.

This study has several limitations. First, it was a retrospective, single-center study, which may limit the generalizability of the model and introduce a potential risk of overfitting. Therefore, multicenter external validation is warranted in future studies to further confirm the robustness and clinical applicability of the model. Second, although B-mode ultrasound is the most widely used modality in clinical practice, enhancing the generalizability and real-world applicability of our model, advanced techniques such as contrast-enhanced ultrasound and shear-wave elastography were not included. These modalities provide additional functional information, including microvascular perfusion and tissue stiffness. Future studies incorporating multiparametric ultrasound data are warranted to further improve model performance and interpretability. Finally, ROI segmentation was performed manually, which may introduce inter-observer variability and limit reproducibility. Although segmentation was conducted independently by two radiologists with consensus review, this process remains time-consuming and operator-dependent. Future studies incorporating automated or deep learning-based segmentation methods may further improve model robustness and facilitate clinical translation.

## Conclusion

In conclusion, this study developed and validated a hierarchical model integrating radiomics features with ALN classification for the preoperative prediction of nodal metastasis and burden in breast cancer. The combined model demonstrated improved predictive performance compared with radiomics alone, highlighting the complementary value of tumor heterogeneity and lymph node morphological features. This approach provides a noninvasive and clinically practical tool for assessing axillary status, which may facilitate risk stratification, guide individualized treatment decisions, and potentially reduce unnecessary invasive procedures.

## Data Availability

The datasets used and/or analyzed during the current study are available from the corresponding author on reasonable request.
