# Supplementary material for: A multimodal ultrasound-based model combining tumor radiomics and axillary lymph node morphologic classification for predicting axillary nodal burden in breast cancer
Source: Front Endocrinol (Lausanne). 2026 Jun 4;17:1847368. doi: 10.3389/fendo.2026.1847368 (PMC13275246; doi:10.3389/fendo.2026.1847368)
Supplement: Supplementary file 1 [file DataSheet1.docx]

**Table S1. Baseline characteristics stratified by nodal tumor burden (N_1–2_ vs. N_≥3_) in the training and testing cohorts**

|  |  | Train (N=150) | | | Test (N=65) | | |
| --- | --- | --- | --- | --- | --- | --- | --- |
|  |  | N_1-2_ (N=76) | N_≥3_ (N=74) | p | N_1-2_ (N=30) | N_≥3_ (N=35) | p |
| Age |  | 55.0 ± 10.3 | 54.2 ± 10.6 | 0.664 | 52.5 ± 9.3 | 52.1 ± 9.8 | 0.873 |
| Molecular subtype |  |  |  | 0.973 |  |  | 0.265 |
|  | Luminal A | 17 (22.4%) | 14 (18.9%) |  | 10 (33.3%) | 10 (28.6%) |  |
|  | Luminal B1 | 19 (25%) | 18 (24.3%) |  | 6 (20%) | 7 (20%) |  |
|  | Luminal B2 | 25 (32.9%) | 27 (36.5%) |  | 10 (33.3%) | 10 (28.6%) |  |
|  | HER2 overexpression | 7 (9.2%) | 8 (10.8%) |  | 1 (3.3%) | 7 (20%) |  |
|  | Triple-Negative | 8 (10.5%) | 7 (9.5%) |  | 3 (10%) | 1 (2.9%) |  |
| ER |  | 0.6 ± 0.4 | 0.5 ± 0.4 | 0.130 | 0.7 ± 0.4 | 0.6 ± 0.4 | 0.292 |
| PR |  | 0.5 ± 0.4 | 0.4 ± 0.4 | 0.042 | 0.5 ± 0.4 | 0.4 ± 0.4 | 0.643 |
| HER2 |  |  |  | 0.417 |  |  | 0.167 |
|  | 0 | 35 (46.1%) | 34 (45.9%) |  | 15 (50%) | 9 (25.7%) |  |
|  | 1 | 11 (14.5%) | 5 (6.8%) |  | 4 (13.3%) | 9 (25.7%) |  |
|  | 2 | 18 (23.7%) | 19 (25.7%) |  | 6 (20%) | 12 (34.3%) |  |
|  | 3 | 12 (15.8%) | 16 (21.6%) |  | 5 (16.7%) | 5 (14.3%) |  |
| Ki-67 |  | 0.4 ± 0.2 | 0.5 ± 0.2 | 0.007 | 0.4 ± 0.2 | 0.4 ± 0.2 | 0.835 |
| BI-RADS |  |  |  | 0.004 |  |  | 0.310 |
|  | 3 | 2 (2.6%) | 1 (1.4%) |  | 4 (13.3%) | 1 (2.9%) |  |
|  | 4a | 3 (3.9%) | 0 (0%) |  | 3 (10%) | 1 (2.9%) |  |
|  | 4b | 15 (19.7%) | 2 (2.7%) |  | 3 (10%) | 6 (17.1%) |  |
|  | 4c | 30 (39.5%) | 42 (56.8%) |  | 12 (40%) | 14 (40%) |  |
|  | 5 | 26 (34.2%) | 29 (39.2%) |  | 8 (26.7%) | 13 (37.1%) |  |
| Lesions size |  |  |  |  |  |  |  |
|  | Length | 2.4 ± 1.1 | 2.9 ± 1.3 | 0.010 | 2.5 ± 1.0 | 2.4 ± 1.3 | 0.899 |
|  | Width | 1.6 ± 0.7 | 1.9 ± 0.9 | 0.044 | 1.7 ± 0.8 | 1.7 ± 1.0 | 0.803 |
| ALN size |  |  |  |  |  |  |  |
|  | Length | 1.2 ± 1.1 | 1.7 ± 1.1 | 0.001 | 1.0 ± 1.0 | 1.6 ± 0.9 | 0.033 |
|  | Width | 0.6 ± 0.6 | 0.9 ± 0.6 | <0.001 | 0.5 ± 0.5 | 0.9 ± 0.5 | 0.012 |
| GTC |  |  |  | 0.600 |  |  | 1.000 |
|  | 1 | 46 (60.5%) | 39 (52.7%) |  | 21 (70%) | 22 (62.9%) |  |
|  | 2 | 20 (26.3%) | 27 (36.5%) |  | 7 (23.3%) | 12 (34.3%) |  |
|  | 3 | 7 (9.2%) | 6 (8.1%) |  | 2 (6.7%) | 1 (2.9%) |  |
|  | 4 | 3 (3.9%) | 2 (2.7%) |  | 0 (0%) | 0 (0%) |  |
| ALN Type |  |  |  | <0.001 |  |  | 0.144 |
|  | 0 | 29 (38.2%) | 9 (12.2%) |  | 11 (36.7%) | 4 (11.4%) |  |
|  | 1 | 2 (2.6%) | 0 (0%) |  | 1 (3.3%) | 1 (2.9%) |  |
|  | 2 | 3 (3.9%) | 1 (1.4%) |  | 1 (3.3%) | 1 (2.9%) |  |
|  | 3 | 7 (9.2%) | 2 (2.7%) |  | 3 (10%) | 1 (2.9%) |  |
|  | 4 | 3 (3.9%) | 1 (1.4%) |  | 1 (3.3%) | 2 (5.7%) |  |
|  | 5 | 11 (14.5%) | 22 (29.7%) |  | 2 (6.7%) | 8 (22.9%) |  |
|  | 6 | 21 (27.6%) | 39 (52.7%) |  | 11 (36.7%) | 18 (51.4%) |  |
| Lesion position |  |  |  |  |  |  |  |
| The lateral breast |  |  |  | 0.358 |  |  | 0.779 |
|  | involved | 64 (84.2%) | 67 (90.5%) |  | 24 (80%) | 30 (85.7%) |  |
|  | uninvolved | 12 (15.8%) | 7 (9.5%) |  | 6 (20%) | 5 (14.3%) |  |
| The medial breast |  |  |  | 0.825 |  |  | 1.000 |
|  | involved | 28 (36.8%) | 25 (33.8%) |  | 10 (33.3%) | 12 (34.3%) |  |
|  | uninvolved | 48 (63.2%) | 49 (66.2%) |  | 20 (66.7%) | 23 (65.7%) |  |
| The inferior breast |  |  |  | 0.050 |  |  | 0.174 |
|  | involved | 25 (32.9%) | 37 (50%) |  | 18 (60%) | 14 (40%) |  |
|  | uninvolved | 51 (67.1%) | 37 (50%) |  | 12 (40%) | 21 (60%) |  |
| The superior breast |  |  |  | 1.000 |  |  | 0.351 |
|  | involved | 64 (84.2%) | 63 (85.1%) |  | 22 (73.3%) | 30 (85.7%) |  |
|  | uninvolved | 12 (15.8%) | 11 (14.9%) |  | 8 (26.7%) | 5 (14.3%) |  |
| CBC history |  |  |  | 0.653 |  |  | 0.891 |
|  | yes | 74 (97.4%) | 70 (94.6%) |  | 28 (93.3%) | 34 (97.1%) |  |
|  | no | 2 (2.6%) | 4 (5.4%) |  | 2 (6.7%) | 1 (2.9%) |  |

**Note:** Data are presented as mean ± standard deviation or number (percentage). N1–2, patients with low nodal tumor burden (1–2 metastatic lymph nodes); N≥3, patients with high nodal tumor burden (≥3 metastatic lymph nodes); ER, estrogen receptor; PR, progesterone receptor; HER2, human epidermal growth factor receptor 2; BI-RADS, Breast Imaging Reporting and Data System; ALN, axillary lymph node; GTC, glandular tissue composition; CBC, contralateral breast cancer. P values were calculated using the independent-samples t-test for continuous variables or the chi-square test for categorical variables, comparing the ALN-negative and ALN-positive groups.

**Table S2. Univariable and multivariable logistic regression analyses of clinical and ultrasound features for predicting axillary lymph node metastasis at the first level**

|  |  | OR (univariable) | OR (multivariable) | OR (final) |
| --- | --- | --- | --- | --- |
| Age |  | 0.99 (0.97-1.01, p=.474) |  |  |
| AUS report | 0 |  |  |  |
|  | 1 | 16.73 (10.04-27.87, p<.001) | 1.45 (0.50-4.20, p=.497) |  |
| BI-RADS | 3 |  |  |  |
|  | 4a | 0.26 (0.07-0.95, p=.041) | 0.23 (0.05-0.98, p=.046) |  |
|  | 4b | 0.74 (0.25-2.20, p=.584) | 0.75 (0.22-2.55, p=.649) |  |
|  | 4c | 1.76 (0.64-4.80, p=.272) | 0.62 (0.19-2.02, p=.432) |  |
|  | 5 | 3.43 (1.20-9.81, p=.021) | 0.73 (0.19-2.73, p=.639) |  |
|  | 6 | 0.93 (0.14-6.23, p=.943) | 0.10 (0.00-2.54, p=.164) |  |
| Lesions size |  |  |  |  |
|  | Length | 1.37 (1.17-1.62, p<.001) | 0.79 (0.50-1.25, p=.311) |  |
|  | Width | 1.79 (1.35-2.37, p<.001) | 1.22 (0.61-2.42, p=.578) |  |
| ALN size |  |  |  |  |
|  | Length | 2.63 (2.09-3.32, p<.001) | 2.13 (0.70-6.50, p=.184) |  |
|  | Width | 13.81 (7.99-23.86, p<.001) | 0.45 (0.04-4.94, p=.512) |  |
| GTC | 1 |  |  |  |
|  | 2 | 1.13 (0.72-1.75, p=.599) |  |  |
|  | 3 | 0.78 (0.38-1.64, p=.517) |  |  |
|  | 4 | 0.76 (0.23-2.53, p=.649) |  |  |
| ALN Type | 0 |  |  |  |
|  | 1 | 1.58 (0.32-7.96, p=.576) | 0.46 (0.04-5.37, p=.537) | 1.60 (0.31-8.16, p=.574) |
|  | 2 | 0.75 (0.27-2.05, p=.574) | 0.32 (0.04-2.36, p=.264) | 0.78 (0.28-2.14, p=.625) |
|  | 3 | 2.96 (1.16-7.53, p=.023) | 1.66 (0.30-9.13, p=.563) | 3.18 (1.23-8.23, p=.017) |
|  | 4 | 3.08 (0.97-9.77, p=.056) | 0.93 (0.12-7.40, p=.948) | 2.74 (0.84-8.89, p=.093) |
|  | 5 | 80.41 (18.30-353.25, p<.001) | 24.07 (2.10-275.93, p=.011) | 70.74 (16.00-312.78, p<.001) |
|  | 6 | 129.39 (38.46-435.37, p<.001) | 60.24 (5.51-658.96, p<.001) | 123.37 (36.52-416.82, p<.001) |
| Lesion position |  |  |  |  |
| The lateral breast | 0 |  |  |  |
|  | 1 | 2.35 (1.37-4.01, p=.002) | 1.68 (0.67-4.19, p=.269) | 1.82 (0.87-3.80, p=.113) |
| The medial breast | 0 |  |  |  |
|  | 1 | 0.58 (0.38-0.87, p=.009) | 0.78 (0.37-1.66, p=.516) |  |
| The inferior breast | 0 |  |  |  |
|  | 1 | 2.12 (1.38-3.26, p<.001) | 1.59 (0.82-3.08, p=.168) | 1.62 (0.87-3.01, p=.128) |
| The superior breast | 0 |  |  |  |
|  | 1 | 0.69 (0.38-1.25, p=.217) |  |  |
| CBC history | 0 |  |  |  |
|  | 1 | 2.31 (0.79-6.80, p=.127) |  |  |

Abbreviations: OR, odds ratio; CI, confidence interval; ALN, axillary lymph node; BI-RADS, Breast Imaging Reporting and Data System; GTC, glandular tissue composition.

**Table S3. Univariable and multivariable logistic regression analyses of clinicopathological features for predicting axillary lymph node metastasis at the first level**

|  |  | OR (univariable) | OR (multivariable) | OR (final) |
| --- | --- | --- | --- | --- |
| Molecular subtype | Luminal A |  |  |  |
|  | Luminal B1 | 3.09 (1.66-5.72, p<.001) | 1.89 (0.92-3.85, p=.082) | 1.89 (0.92-3.85, p=.082) |
|  | Luminal B2 | 1.65 (0.97-2.79, p=.063) | 1.09 (0.59-2.03, p=.779) | 1.09 (0.59-2.03, p=.779) |
|  | HER2 overexpression | 1.57 (0.76-3.27, p=.223) | 0.70 (0.26-1.87, p=.474) | 0.70 (0.26-1.87, p=.474) |
|  | Triple-Negative | 1.64 (0.78-3.41, p=.189) | 0.62 (0.22-1.76, p=.368) | 0.62 (0.22-1.76, p=.368) |
| ER |  | 0.65 (0.39-1.07, p=.092) |  |  |
| PR |  | 0.57 (0.33-0.97, p=.036) | 0.56 (0.27-1.17, p=.124) | 0.56 (0.27-1.17, p=.124) |
| HER2 | 0 |  |  |  |
|  | 1 | 1.11 (0.60-2.05, p=.738) |  |  |
|  | 2 | 1.01 (0.61-1.67, p=.975) |  |  |
|  | 3 | 1.01 (0.57-1.79, p=.961) |  |  |
| Ki-67 |  | 4.02 (1.73-9.37, p=.001) | 3.42 (1.18-9.93, p=.024) | 3.42 (1.18-9.93, p=.024) |

Abbreviations: OR, odds ratio; CI, confidence interval; ER, estrogen receptor; PR, progesterone receptor; HER2, human epidermal growth factor receptor 2.

**Table S4. Univariable and multivariable logistic regression analyses of clinical and ultrasound features for predicting nodal tumor burden at the second level**

|  |  | OR (univariable) | OR (multivariable) | OR (final) |
| --- | --- | --- | --- | --- |
| Age |  | 0.99 (0.96-1.02, p=.661) |  |  |
| BI-RADS | 3 |  |  |  |
|  | 4a | 0.00 (0.00-Inf, p=.986) |  |  |
|  | 4b | 0.27 (0.02-4.46, p=.358) |  |  |
|  | 4c | 2.80 (0.24-32.31, p=.409) |  |  |
|  | 5 | 2.23 (0.19-26.06, p=.522) |  |  |
| Lesions size |  |  |  |  |
|  | Length | 1.42 (1.08-1.86, p=.012) | 1.48 (0.86-2.54, p=.157) |  |
|  | Width | 1.55 (1.00-2.39, p=.050) | 0.66 (0.28-1.57, p=.347) |  |
| ALN size |  |  |  |  |
|  | Length | 1.64 (1.20-2.24, p=.002) | 0.93 (0.40-2.19, p=.868) |  |
|  | Width | 2.81 (1.51-5.23, p=.001) | 0.99 (0.17-5.77, p=.995) |  |
| GTC | 1 |  |  |  |
|  | 2 | 1.59 (0.78-3.27, p=.204) |  |  |
|  | 3 | 1.01 (0.31-3.26, p=.985) |  |  |
|  | 4 | 0.79 (0.12-4.95, p=.798) |  |  |
| ALN Type | 0 |  |  |  |
|  | 1 | 0.00 (0.00-Inf, p=.989) | 0.00 (0.00-Inf, p=.989) | 0.00 (0.00-Inf, p=.989) |
|  | 2 | 1.07 (0.10-11.65, p=.953) | 1.18 (0.08-16.57, p=.902) | 1.20 (0.11-13.24, p=.884) |
|  | 3 | 0.92 (0.16-5.25, p=.926) | 1.18 (0.16-8.71, p=.873) | 1.12 (0.19-6.56, p=.900) |
|  | 4 | 1.07 (0.10-11.65, p=.953) | 1.67 (0.12-22.57, p=.700) | 1.20 (0.11-13.24, p=.884) |
|  | 5 | 6.44 (2.28-18.25, p<.001) | 6.00 (1.27-28.38, p=.024) | 6.10 (2.13-17.43, p<.001) |
|  | 6 | 5.98 (2.39-14.97, p<.001) | 7.36 (1.60-33.79, p=.010) | 6.28 (2.48-15.92, p<.001) |
| Lesion position |  |  |  |  |
| The lateral breast | 0 |  |  |  |
|  | 1 | 1.79 (0.66-4.84, p=.248) |  |  |
| The medial breast | 0 |  |  |  |
|  | 1 | 0.87 (0.45-1.71, p=.695) |  |  |
| The inferior breast | 0 |  |  |  |
|  | 1 | 2.04 (1.05-3.95, p=.034) | 1.87 (0.85-4.08, p=.118) | 1.80 (0.86-3.78, p=.117) |
| The superior breast | 0 |  |  |  |
|  | 1 | 1.07 (0.44-2.61, p=.875) |  |  |
| CBC history | 0 |  |  |  |
|  | 1 | 2.11 (0.38-11.91, p=.396) |  |  |

Abbreviations: OR, odds ratio; CI, confidence interval; ALN, axillary lymph node; GTC, glandular tissue composition.

**Table S5. Univariable and multivariable logistic regression analyses of clinicopathological features for predicting nodal tumor burden at the second level**

|  |  | OR (univariable) | OR (multivariable) | OR (final) |
| --- | --- | --- | --- | --- |
| Molecular subtype | Luminal A |  |  |  |
|  | Luminal B1 | 3.09 (1.66-5.72, p<.001) | 1.89 (0.92-3.85, p=.082) | 1.89 (0.92-3.85, p=.082) |
|  | Luminal B2 | 1.65 (0.97-2.79, p=.063) | 1.09 (0.59-2.03, p=.779) | 1.09 (0.59-2.03, p=.779) |
|  | HER2 overexpression | 1.57 (0.76-3.27, p=.223) | 0.70 (0.26-1.87, p=.474) | 0.70 (0.26-1.87, p=.474) |
|  | Triple-Negative | 1.64 (0.78-3.41, p=.189) | 0.62 (0.22-1.76, p=.368) | 0.62 (0.22-1.76, p=.368) |
| ER |  | 0.65 (0.39-1.07, p=.092) |  |  |
| PR |  | 0.57 (0.33-0.97, p=.036) | 0.56 (0.27-1.17, p=.124) | 0.56 (0.27-1.17, p=.124) |
| HER2 | 0 |  |  |  |
|  | 1 | 1.11 (0.60-2.05, p=.738) |  |  |
|  | 2 | 1.01 (0.61-1.67, p=.975) |  |  |
|  | 3 | 1.01 (0.57-1.79, p=.961) |  |  |
| Ki-67 |  | 4.02 (1.73-9.37, p=.001) | 3.42 (1.18-9.93, p=.024) | 3.42 (1.18-9.93, p=.024) |

Abbreviations: OR, odds ratio; CI, confidence interval; ER, estrogen receptor; PR, progesterone receptor; HER2, human epidermal growth factor receptor 2.


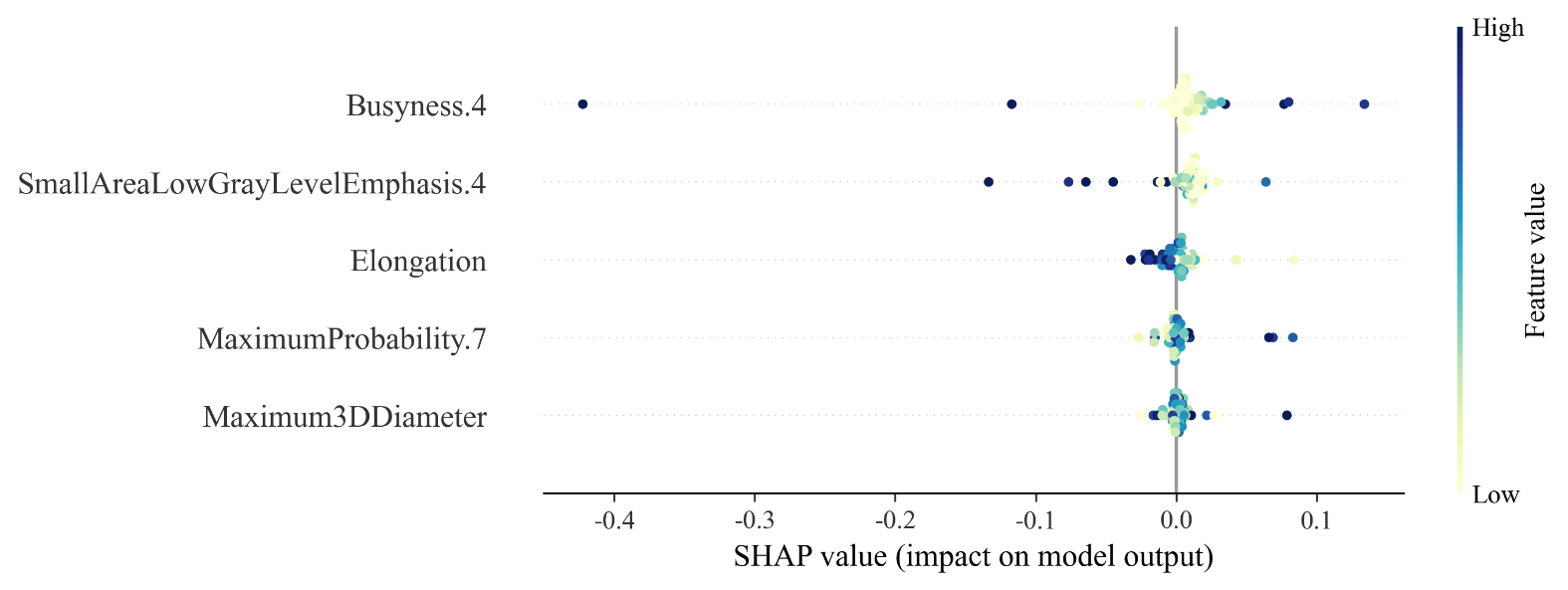


**FigureS1. SHAP summary plot of the second-level model for predicting axillary nodal tumor burden (N_1–2_ vs. N_≥3_).**

Each point represents an individual sample. The x-axis indicates the SHAP value (impact on model output), and the color denotes the feature value (low to high). Features are ranked in descending order of importance.
